# Perioperative Bottom‐Up vs. Top‐Down Attention Impairments in Postoperative Delirium

**DOI:** 10.1002/alz70861_108129

**Published:** 2025-12-23

**Authors:** Melody R. Smith, Kenneth C Roberts, Jeffrey N. Browndyke, Mary Cooter Wright, Tyler H. Reekes, Michael J. Devinney, Harvey Jay Cohen, Joseph P. Mathew, Heather Whitson, Marty G. Woldorff, Miles Berger

**Affiliations:** ^1^ Stanford University, Charlotte, NC USA; ^2^ Duke University Medical Center, Durham, NC USA; ^3^ Duke Center for the Study of Aging and Human Development, Durham, NC USA; ^4^ Duke University, Durham, NC USA; ^5^ Duke Brain Imaging & Analysis Center, Durham, NC USA; ^6^ Duke Institute for Brain Sciences, Durham, NC USA; ^7^ Durham VA Medical Center, Durham, NC USA; ^8^ Department of Psychiatry and Behavioral Sciences, Duke University Medical Center, Durham, NC USA; ^9^ Anesthesiology Department, Duke University School of Medicine, Durham, NC USA; ^10^ Duke University Department of Anesthesiology, Durham, NC USA; ^11^ Duke/UNC Alzheimer’s Disease Research Center, Duke University School of Medicine, Durham, NC USA; ^12^ Duke University School of Medicine, Durham, NC USA; ^13^ Center for Cognitive Neuroscience & Duke Institute for Brain Sciences (DIBS), Duke University, Durham, NC USA; ^14^ Stanford University Department of Anesthesiology, Perioperative and Pain Medicine, Palo Alto, CA USA; ^15^ Center for the Study of Aging & Human Development, Duke University, Durham, NC USA; ^16^ Duke University Center for Cognitive Neuroscience, Durham, NC USA

## Abstract

**Background:**

Postoperative delirium (POD), characterized by acute fluctuations in consciousness and attention, is associated with increased risk of Alzheimer's disease and related dementias. However, the neural mechanisms underlying attention impairments in POD are not well‐understood. The dorsal attention network (DAN) controls voluntary, top‐down attention processing via the superior parietal lobules, intraparietal sulci, and frontal eye fields, while the ventral attention network (VAN) controls automatic, bottom‐up processing via the right temporoparietal junction and ventral frontal cortices. Both networks are activated during the Posner paradigm. Prior studies suggest that older adults require compensatory brain resources to focus on attentional Posner tasks, potentially due to age‐related declines in attentional network function. It is unknown whether attention network impairments among older adults would increase risk for POD. Conversely, it is unknown whether POD contributes to or accelerates attention network impairments among older adults after surgery. This work expands this limited literature by studying attention network impairments among older surgical patients with and without POD.

**Method:**

Interim data included 143 non‐cardiac, non‐neurologic surgical patients aged ≥65 with a planned ≥2‐hour non‐cardiac, non‐neurologic surgery (from NIH R01‐AG073598, ALADDIN). Posner performance was assessed before surgery and days 1‐7 after surgery or until hospital release. POD was assessed via 3D‐CAM twice daily from day of surgery to postoperative day 7 or hospital release.

**Result:**

Repeated measures analyses (group, time, group‐by‐time) demonstrated that, preoperatively, patients who did (vs. did not) develop POD had lower accuracy (β[95% CI]: ‐0.07[‐0.12 ‐ ‐0.02], *p* =0.005) and lower response rates (‐0.05[‐0.10 ‐ ‐0.01], *p* =0.021) for Posner trials requiring top‐down attention processing. From preoperative to postoperative timepoints, patients who did (vs. did not) develop POD had greater deterioration in top‐down reaction times (45.5 [12.8 – 78.2], *p* =0.007), bottom‐up accuracy (‐0.13[‐0.20 ‐ ‐0.05], *p* =0.001) and bottom‐up response rates (‐0.11[‐0.18 ‐ ‐0.04], *p* =0.003).

**Conclusion:**

Among older surgical patients, attention impairments associated with POD manifest preoperatively and postoperatively as distinct patterns, namely preoperative impairments in top‐down (DAN‐regulated) attention processing and postoperative deterioration in top‐down (DAN‐regulated) and bottom‐up (VAN‐regulated) processing. Future research should combine the Posner paradigm with EEG/MEG/MRI to pinpoint the source of these POD impairments.

